# Accumulation of α-2,6-sialyoglycoproteins in the Muscle Sarcoplasm Due to *Trichinella Sp*. Invasion

**DOI:** 10.1515/biol-2019-0053

**Published:** 2019-12-18

**Authors:** Rositsa Milcheva, Pavol Janega, Peter Celec, Svetlozara Petkova, Zuzana Hurniková, Barbora Izrael-Vlková, Katerina Todorova, Pavel Babál

**Affiliations:** 1 Department of Pathology, IEMPAM, Bulgarian Academy of Sciences, ‘’Acad. G. Bonchev’’ Str. 25, 1113, Sofia, Bulgaria; 2 Department of Pathology, Faculty of Medicine, Comenius University in Bratislava, Sasinkova 4, 81372 Bratislava, Slovakia; 3 Department of Molecular Biomedicine, Faculty of Medicine, Comenius University in Bratislava, Sasinkova 4, 81372 Bratislava, Slovakia; 4 Institute of Experimental Morphology, Pathology and Anthropology with Museum (IEMPAM), Bulgarian Academy of Sciences, “Acad. G. Bonchev” Str. 25, 1113 Sofia, Bulgaria; 5 Institute of Parasitology, Slovak Academy of Sciences, Hlinkova 3, 040 01 Košice, Slovak Republic

**Keywords:** Nurse cell, sialic acids, skeletal muscle, *Trichinella*

## Abstract

The sialylation of the glycoproteins in skeletal muscle tissue is not well investigated, even though the essential role of the sialic acids for the proper muscular function has been proven by many researchers. The invasion of the parasitic nematode *Trichinella spiralis* in the muscles with subsequent formation of Nurse cell-parasite complex initiates increased accumulation of sialylated glycoproteins within the affected area of the muscle fiber. The aim of this study is to describe some details of the α-2,6-sialylation in invaded muscle cells. Asynchronous invasion with infectious *T. spiralis* larvae was experimentally induced in mice. The areas of the occupied sarcoplasm were reactive towards α-2,6-sialic acid specific *Sambucus nigra* agglutinin during the whole process of transformation to a Nurse cell.The cytoplasm of the developing Nurse cell reacted with *Helix pomatia* agglutinin, *Arachis hypogea* agglutinin and *Vicia villosa* lectin-B4 after neuraminidase pretreatment.Up-regulation of the enzyme ST6GalNAc1 and down-regulation of the enzyme ST6GalNAc3 were detected throughout the course of this study. The results from our study assumed accumulation of sialyl-Tn-Ag, 6`-sialyl lactosamine, SiA-α-2,6-Gal-β-1,3-GalNAc-α-Ser/Thr and Gal-β-1,3-GalNAc(SiA-α-2,6-)-α-1-Ser/Thr oligosaccharide structures into the occupied sarcoplasm. Further investigations in this domain will develop the understanding about the amazing adaptive capabilities of skeletal muscle tissue.

## 1 Introduction

Among all known myopathies, the establishment of a Nurse cell-parasite complex resulting from infestation by the parasitic nematode *Trichinella* is a unique event. This structure derives from a portion of the striated skeletal muscle fiber and develops within 15 to 20 days after a larva of *Trichinella* invades the fiber [1]. After penetrating the skeletal muscle fiber, the larva induces morphological, functional and enzymatic changes. The occupied portion of the muscle fiber transforms into a structure called Nurse cell, capable of supporting the parasite for years [1]. During this process of de-differentiation, at least 53 genes associated with apoptosis, satellite cell activation and proliferation, cell differentiation, cell proliferation and cycle regulation, myogenesis and muscle development change in expression [2]. The affected areas lose their contractile properties but the membranes of the newly developing Nurse cells remain adherent within the construction of the contractile fiber.

Sialic acids represent more than 40 modifications of the nine-carbon N-acetylneuraminic acid (NANA), which is synthesized from N-acetylmanosamine-6-phosphate and phosphoenolpyruvate in the cytosol. The molecule of NANA is activated in the nucleus by the transfer of a cytidinemonophosphate residue (CMP) and then is translocated to the Golgi apparatus or the endoplasmic reticulum. There, the activated sialic acid can be transferred by a sialyltransferase onto anappropriate acceptor molecule from the oligosaccharide chain of a nascent glycoconjugate via alpha-2,3, -2,6 or -2,8 glycosidic bond[3]. With their outer position on the oligosaccharide chains, the sialic acids are involved in almost all types of recognition phenomena and adhesion mechanisms, either through masking sites of biological recognition or by representing recognition epitopes [4, 5]. They also have a crucial role in the process of gene expression and cell differentiation [6]. The great diversity of oligosaccharide structures,which are destined to be sialylated, determines the abundance of the existing highly specific enzymes sialyltransferases. What determines their specificity is that the recognition of a sugar residue as a suitable receptor often depends on the construction of the entire oligosaccharide chain. Four families of sialyltransferases have been identified by now. The members of the families ST3Gal, ST6Gal and ST6GalNAc are broadly distributed in different tissues, and the enzymes from the ST8SiA family are predominantly expressed in the brain [7].

In skeletal muscles, the sialic acids are important for the functional maintenance of glycoproteins involved in fiber structure and neuromuscular junctions [8, 9], development and regeneration [10], muscle excitability [11, 12], exercise performance [13] and reactive oxygen species scavenging [14]. Considering this role of sialic acids in adhesive mechanisms and proper function of the skeletal muscle tissue, we hypothesized that the process of transformation into a Nurse cell following to invasion by *Trichinella*, should be associated with an increased sialylation of the affected fibers. Previously, we showed that the presence of *Trichinella spiralis* in the muscles initiates glycosylation changes with increased sialic acid synthesis within the affected area of the muscle fiber [15].

The present work describes the changes in expression of α-2,6-sialylated glycoproteins and sialyltransferases in mouse skeletal muscles after invasion by *T. spiralis*.

## 2 Material and Methods

### 2.1 Ethical procedures

All animal experiments were performed in compliance with the Institutional Guidelines for Animal Experiments of the Faculty of Medicine, Comenius University in Bratislava, of the Institute of Parasitology, Slovak Academy of Sciences, and of the Institute of Experimental morphology, Pathology and Anthropology with Museum, Bulgarian Academy of Sciences, following the EU and locally established norms and procedures.

**Ethical approval**: The research related to animals use has been complied with all the relevant national regulations and institutional policies for the care and use of animals.

### 2.2 Parasites, invasion, sample collection and tissue preparation for basic pathomorphology

Infective *T. spiralis* larvae were isolated from previously invaded mice, between30 and 40 days post infection (d.p.i.) according to a routine protocol [16]. Crl:CD1 (ICR) mice (Velaz, Prague, Czech Republic), 6-8 weeks old, were inoculated with 500 infective *T. spiralis* larvae *per os*. The animals (five per group) were sacrificed at day 0, 10, 14, 18, 25 and 40 post invasion. Tissue specimens were excised from the femoral, pectoral and gluteal muscles and fixed with freshly prepared modified methacarn fixative [17], or stored at -80ºC for further molecular and proteomic studies. After processing, the specimens were embedded in paraffin.

### 2.3 Preparation of rabbit hyper-immune serum against L1 larvae of *T. spiralis*

Crude antigen (Ag) of muscle *T. spiralis* larvae and the rabbit hyper-immune serum against infectious larvae of *T. spiralis* were prepared as already described [15, 18].

### 2.4 Histochemistry

All reagents and standard procedures for lectin and immunohistochemistry were previously described in detail [15]. Tissue sections, 5 μm thick, from all experimental groups were submitted for staining with hematoxylin and eosin (H&E) for basic morphological evaluation (minimum five sections *per* mouse), and for immunohistochemistry and lectin histochemistry (minimum seven sections *per* mouse).

#### 2.4.1 Immunochistochemistry

For verification of the sites of occupation, tissue sections were treated for 16 hours with rabbit hyper-immune serum against infectious larvae of *T. spiralis* (1:5000), then incubated for 30 min with EnVision^TM^anti-rabbit polymer conjugated with horseradish peroxidase (Dako) and the peroxidase activity was developed with EnVision^TM^ 3,3`-diaminobenzidine (DAB+) Substrate-Chromogen (Dako).

#### 2.4.2 Lectin histochemistry

The tissue sections were divided in groups according to the applied lectin and each group included experimental sections, negative controls, sections incubated with previously blocked lectin and neuraminidase pre-treated sections. The carbohydrate specificity of the lectins used in this study is listed in Table 1. The sections were incubated with *Sambucus nigra* agglutinin (SNA, 1 μg/ml), *Vicia villosa* lectin-B4 (VVL-B4, 6 μg/ml, Vector, Burlingame CA, USA), Peanut agglutinin (PNA, 10 μg/ml) and *Helix pomatia* agglutinin (HPA, 10 μg/ml, Sigma Aldrich, Saint Louis, USA) for 1 hour at room temperature. The binding specificities were tested by pre-incubation with 0.1M solutions of target monosaccharides for 1 hour at room temperature: 6`-Sialyl Lactose (Calbiochem-Novabiochem) for SNA, α-D-galactose-1-phosphate, dipotassium salt for HPA, PNA and VVL, N-acetyl-D-galactosamine for HPA and VVL-B4, and D-galactose (Sigma-Aldrich) for PNA. Pre-incubation of HPA, PNA and VVL-B4 was intended only for neuraminidase pretreated sections. The neuraminidase pre-treatment was performed with 0.5U/mL of neuraminidase from *Clostridium perfringens* (Sigma-Aldrich) in 0.05 M sodium acetate buffer (pH 5.5), at 37^⍛^C for 15 hours before application of peroxide and biotin blocking systems. In parallel, negative control specimens were incubated with tris buffered saline (TBS) instead of lectin. All of the sections were then treated with Streptavidin-HRP (Dako) for 30 min and the peroxidase activity was developed with DAB+ (Dako).

**Table 1:** The lectins used in this study, their abbreviations, carbohydrate specificity, and related references.

Lectin	Abbreviation	Carbohydrate specificity	References
*Arachis hypogea* agglutinin	PNA	Galβ(1,3)GalNAc	[19]
*Helix pomatia* agglutinin	HPA	GalNAc-α	[20]
*Sambucus nigra* agglutinin	SNA	SiAα(2,6)Galβ(1,4)GlcNAc	[21]
		SiAα(2,6)Galβ(1,3)GalNAc	[22]
		SiAα(2,6)GalNAc-α-O-Ser/Thr	
*Vicia villosa* lectin – isoform B_4_	VVL-B_4_	GalNAc-α-O-Ser/Thr	[23]

SiA –sialic acid, Gal – galactose, GalNAc – N-acetyl-D-galactosamine, GlcNAc – N-acetyl-D-glucosamine, Ser – serine, Thr – threonine.

The sections were examined with Nikon Eclipse 80ί light microscope.

The intensity of the histochemical staining within the occupied fibers was evaluated semi-quantitatively *versus* the surrounding non-affected sarcoplasm by two independent observers into four categories: negative (-),weak (+), moderate (++),andstrong (+++) positive.

### 2.5 Gene expression analyses

#### 2.5.1 Isolation of RNA

Total RNA was extracted from mouse skeletal muscle specimens from all experimental groups using RNazol®RT (Molecular Research Center, Cincinnati, OH, USA) with slight modifications of the provided protocol. In brief, 60 mg tissues were homogenized in 500 μl RNazol®RT and stored overnight at -20^⍛^C. After thawing, 500 μl RNazol®RT and 400 μl DEPC-treated water (Fermentas Thermo Fisher Scientific, Waltham, MA, USA) per ml RNazol®RTwere added. The samples were centrifuged at 15 000 rpm for 8 min, the total RNA from the supernatant was precipitated with 1:1 (v/v) isopropanol and the pellets were dissolved in DEPC-treated water. RNA was quantified by measuring the optical density (OD) at λ=260 nm on a Biophotometer (Eppendorf, Hamburg, Germany) *versus* DEPC-treated water.The samples were stored at -80°C.

#### 2.5.2 Primer design

Primers for amplification of fragments of the reference gene peptidyl prolyl isomerase A (PPIA, accession number NM_008907.1), beta galactoside alpha-2,6-silalyltransferase 1 (ST6Gal1, NM_145933.3) and [alpha-N-acetyl-neuraminyl-2,3-beta-galactosyl-1,3]-N-acetylgalactosaminide alpha-2,6-sialyltransferase type 1 (ST6GalNAc1, NM_011371.2), type 2 (ST6GalNAc2, NM_009180.3) and type 3 (ST6GalNAc3, NM_011372.2) of *Mus musculus* were designed using the NCBI Blast Tool [24] in a way to span at least one intron sequence. The primers and the size of the products are listed in Table 2. The oligonucleotides were purchased from Sigma-Aldrich (Steinheim, Germany).The substrate specificities of all sialyltansferases analysed in this study are listed in Table 3.

**Table 2:** The reverse (R) and the forward (F) primers used in this study.

Gene	Primers sequences (5`-3`)	Product size (bp)
PPIA	ccagtgccattatggcgt - R	115
	ttcgagctctgagcactgg - F	
ST6Gal1	tggaagttgtctgtaggtgcccc – R	107
	gccgtcgtgtcttctgcaggat – F	
ST6GalNAc1	tctcctgggcacttgcgtca – R	117
	tcctgcttctgactgtgttggca - F	
ST6GalNAc2	tcaaacaggctgcggaagcga – R	117
	cccacgagcattctttgaccca - F	
ST6GalNAc3	accttctgcccgaccatttgacc – R	120
	caggcagcctcttcgaactcact – F	

-

**Table 3:** The substrate specificities of the sialyltransferases investigated in this study and the types of the formed glycosidic linkages.

Enzyme (abbreviation)	Substrat specificity	Type of created linkage	References
ST6Gal1	**Gal**-β-1,4-GlcNAc-	α-2,6-	[25]
ST6GalNAc1	**GalNAc**-α-1-Ser/Thr	α-2,6-	[26]
ST6GalNAc2	Gal-β-1,3-**GalNAc**-α-1-Ser/Thr	α-2,6-	[26]
	SiA-α-2,3-Gal-β-1,3-**GalNAc**-α-1-Ser/Thr		
ST6GalNAc3	SiA-α-2,3-Gal-β-1,3**-GalNAc**-	α-2,6-	[27]

SiA –sialic acid, Gal – galactose, GalNAc – N-acetyl-D-galactosamine, GlcNAc – N-acetyl-D-glucosamine, Ser – serine, Thr – threonine. The monosaccharides in bold indicate a residue onto which a SiA is transferred.

#### 2.5.3 Reverse transcription (RT) reaction

Approximately 750 ng RNA from each sample were used for first strand cDNA synthesis. The RT Master Mix contained 5x Reaction Buffer (Fermentas Thermo Fisher Scientific), 20 U RNase Inhibitor (Genecraft, Cologne, Germany), 1 mM dNTPs (Finnzymes Oy, Espoo, Finland), 200 U RevertAid™ M-MuLV Reverse Transcriptase (Fermentas Thermo Fisher Scientific), 100 pmol random hexameres (Fermentas Thermo Fisher Scientific) and DEPC-treated water. The reaction mixture was first incubated at room temperature for 10 min, then at 42°C for 1h, and the reaction was terminated at 70°C for 10 min. The generated cDNA was quantified and the samples were stored at -80°C.

#### 2.5.4 End point PCR

Hot start PCR was designed on approximately 700 ng cDNA as a template in 20 μl total volume of reaction by using PCR Thermocycler (Eppendorf). The PCR Master Mix contained 5xGreen GoTaq®Flexi Buffer (Promega, Madison, WI, USA), 1.5 mM MgCl_2_ (Fermentas Thermo Fisher Scientific), 0.2 mM dNTP mix (Finnzymes Oy), 1.25 U GoTaq®Hot Start Polymerase (Promega), 0.3 μM R- and F-primers specific for amplification of fragments of PPIA, ST6Gal1, ST6GalNAc1, -2, and –3, and DEPC-treated water. Three PCR reactions/group were performed for amplification of each fragment of interest. The products of amplification were visualized on a 2% agarose gel supplemented with GoldView Nucleic acid stain (SBS, Beijing, China) *versus* GeneRuler™ 100 bp Plus DNA Ladder (Fermentas Thermo Fisher Scientific). The gels were scanned in iBOX® 500 UPV Imaging System (Synoptics Ltd, Cambridge, UK) and after calibration of the gels the density of the bands was measured by ImageJ Software (NIH, USA) [28].

Normalization of target gene expressions *versus* the expression of the reference gene was calculated as published by Boonmars et al. [29] and the resulting ratios (in arbitrary units) were plotted against the timeline of the experiment.

#### 2.5.5 Statistical analysis of semiquantitative evaluation of the gene expressions

Statistical evaluation of the data was performed using GraphPad Prism 5.03 software (San Diego, CA, USA). D`Agostino-Pearson test was applied to quantify the normality of the data set. Non-parametric one-way analysis of variance (Kruskal-Wallis test) with Dunn`s Multiple Comparison Test (significance level 0.05) was computed to detect statistically significant differences in the yield of the PCR products between the control and infected samples, and the results were interpreted as follows: P < 0.001 = highly significant, P < 0.01 = very significant, P < 0.05 = significant.

## 3 Results

### 3.1 Histology of the process of de-differentiation of the affected skeletal muscle fibres after invasion by T. spiralis

**Figure 1:**
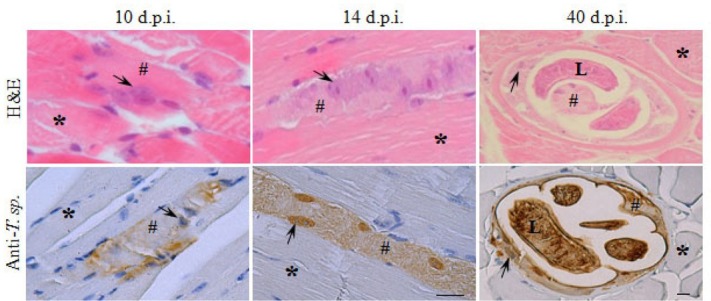
Basic histology and immunohistochemistry on mouse skeletal muscles with *Trichinella spiralis*. Formalin fixed sections, collected at days 10, 14 and 40 post invasion (d.p.i.), werestained by Hematohylin-Eosin (H&E) and with rabbit hyper-immune serum against infectious larvae (anti-*T. sp*). The brown colour indicates positive histochemical reaction; hash tag – occupied sarcoplasm, star – non-occupied skeletal muscle fibre, arrow – enlarged nucleus, L– larva. Scale bar 20 μm.

The occupied sites in skeletal muscle specimens from day 10 p.i. were distinguished by the enlargement and centralization of nuclei of the fiber. After day 14 p.i. the affected cytoplasm of the fibre gradually disintegrated and at 40 d.p.i. the de-differentiation of the occupied fiber into a Nurse cell was complete. The enlarged nuclei were a permanent characteristic of the process of de-differentiation and persisted also within the capsulated Nurse cell containing a coiled larvae. The invaded muscle fibres at all investigated time points were labelled by the rabbit hyper-immune serum against infectious larvae of *T. spiralis* (Fig. 1) indicating the presence of larval excretory-secretory products within the sarcoplasm and the enlarged nuclei. The surrounding non-affected areas remained unstained (Fig. 1).

### 3.2 Lectin histochemistry of healthy and invaded mouse skeletal muscle fiber

Detailed results from the lectin histochemistry are showed in Table 4.

**Table 4:** Evaluation of the intensity of intra-cellular staining in the invaded muscle fibers after lectin-histochemistry.

	Days post infestation				
Lectin	10	14	18	25	40
HPA	-	++	-	-	-
HPA-D-GalNAc	-	-	-	-	-
HPA-Neu	-	++	++	++	++
HPA-Neu-D-GalNAc	-	-	-	-	-
PNA	-	-	-	-	-
PNA-Neu	++	+++	+++	+++	+++
PNA-Neu-D-Gal	-	-	-	-	-
SNA	++	+++	+++	+++	+++
SNA-6`-SiaLac	-	-	-	-	-
SNA-Neu	-	+	++	++	++
VVL-B4	-	-	-	-	-
VVL-B4-Neu	-	++	++	-	-
VVL-B4-Neu-D-GalNAc	-	-	-	-	-

Sections were with and without neuraminidase pretreatment (Neu), or preincubation with N-acetyl-D-galactosamine (D-GalNAc), D-galactose (D-Gal), and 6`-Sialyl Lactose (6`-SiaLac). The results were interpreted as negative (-),weak (+), moderate (++) and strong (+++) positive.

**Figure 2:**
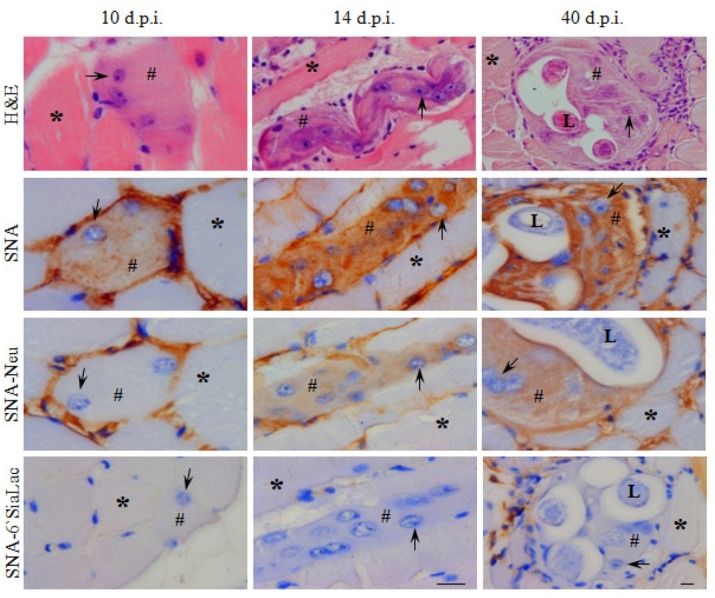
Appearance of α-2,6-sialylation in the sarcoplasm after invasion by *T. spiralis*. Modified methacarn fixed sections from mouse skeletal muscles with *Trichinella spiralis* at days 10, 14 and 40 postinvasion (d.p.i.) were stained with biotinylated lectin SNA, specific for α-2,6-sialic acid. The specificity of the lectin was tested with neuraminidase (Neu) pretreatment of the sections, and with preincubation of the lectin with 6`-sialyl lactose (6`-SiaLac). Parallel sections were subjected to H&E staining to facilitate the histological orientation. The brown colour indicates positive histochemical reaction, the hashtag indicates the occupied sarcoplasma, a star – non-occupied skeletal muscle fibre, and an arrow – enlarged nucleus, L– larva. The scale bar is 20 μm.

**Figure 3:**
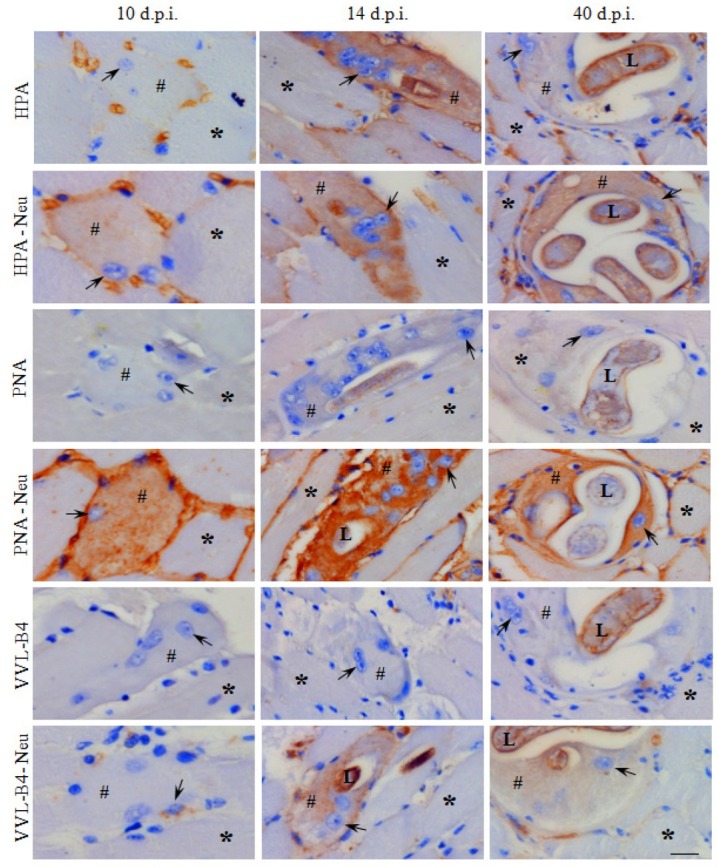
Lectin histochemistry. Modified methacarn fixed sections from mouse skeletal muscles with *Trichinella spiralis* at days 10, 14 and 40 post invasion (d.p.i.) were stained with biotinylated lectins HPA, PNA, and VVL-B4 with and without neuraminidase (Neu) pretreatment. The brown colour indicates positive histochemical reaction, the hashtag indicates the occupied sarcoplasma, a star – non-occupied skeletal muscle fibre, and an arrow – enlarged nucleus, L – larva. The scale bar is 20 μm.

The sarcoplasm of healthy skeletal muscle fibers did not react with any of the lectins used in the study. SNA stained the sarcolemma; after neuraminidase treatment the staining diminished (Fig. 2). HPA stained only the blood vessels; after neuraminidase treatment, HPA and PNA intensely stained the sarcolemma (Fig. 3). The worm did not react with SNA; whereas PNA, HPA and VVL-B4 stained the cuticle, stychosome and hypodermis of *Trichinella* (Fig. 2, 3).

The areas of the occupied sarcoplasm were reactive towards SNA during the whole process of transformation and within the mature Nurse cell (Table 4, Fig. 2). Except for HPA at day 14 p.i., the occupied sarcoplasm was reactive with HPA, PNA and VVL-B4 only after pretreatment of the sections with neuraminidase (Table 4, Fig. 3). With small exceptions (VVL-B4), the intensity of the staining of the sarcoplasm with all of the lectins used in the study increased progressively in the time course of Nurse cell formation (Table 4, Fig. 3).

### 3.3 Expression of sialyltransferases in healthy and invaded mouse skeletal muscle samples

**Figure 4:**
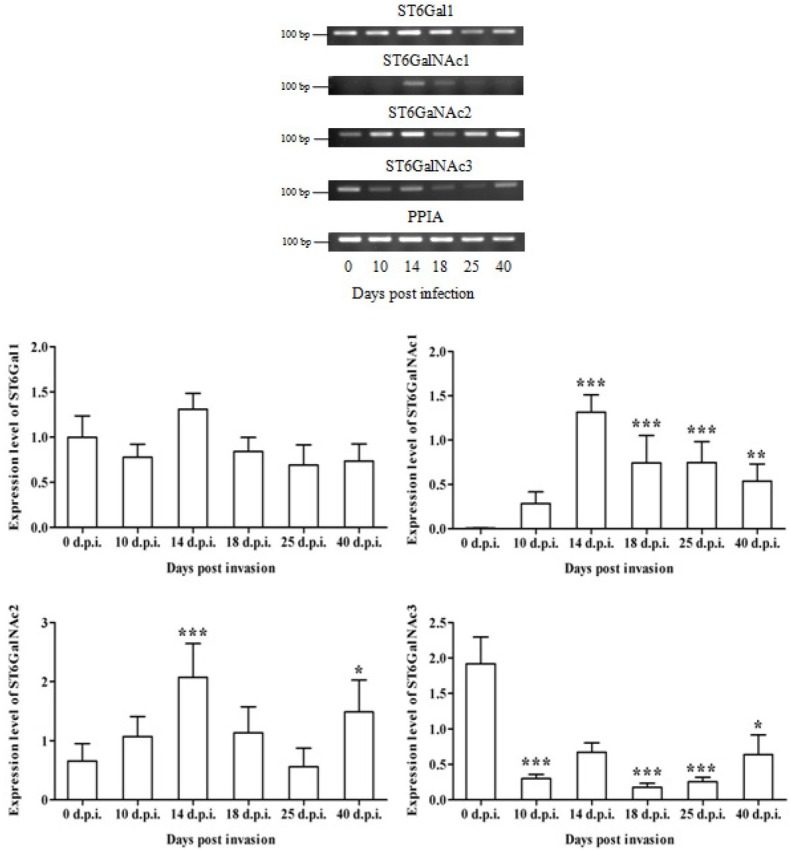
Sialyltransferase expressions in mouse skeletal muscle tissue invaded with *Trichinella spiralis*.

The mRNAs for ST6Gal1, ST6GalNAc2 and ST6GalNAc3 were present in healthy mouse skeletal muscle tissue but expression of ST6GalNAc1 was not detected by end-point PCR (Fig. 4). Expression of ST6GalNAc1 was detected at day 10 p.i. and significantly increased at days 14, 18, 25 and 40 p.i. (Fig. 4). The expressions of ST6GalNAc2 increased at days 14 and 40 p.i. Significant decrease of ST6GalNAc3 expression was observed at days 10, 18, 25 and 40 p.i. The expression of ST6Gal1 remained unchanged (Fig. 4).

The relative mRNA expression of ST6Gal1, ST6GalNAc1, ST6GalNAc2 and ST6GalNAc3sialyltransferases was evaluated by two step RT-PCR. The optical density of product-specific bands was measured by means of ImageJ software. The absolute values (arbitrary units) were normalized *versus* the reference gene PPIA for each sample and calculated ratios were plotted. Nonparametric one-way analysis of variance with Dunn`s Multiple Comparison Test of the ratios of normalization were computed; the error bars represent 95%CI of nine independent normalized ratios. The stars indicate statistically significant difference of mRNA expressions of the invaded tissues in progress *versus* the non-affected skeletal muscle used as a control: *** P < 0.001, ** P< 0.01, * P< 0.05.

The photographs show one representative replicate of 2% agarose gel for each of the five amplicons used in this study.

### 3.4 Predicted sialylated oligosaccharides in the mouse skeletal muscle sarcoplasm after invasion by T. spiralis

An overview of the concluded results is presented in Table 5.

**Table 5:** Summary of the predicted sialylated oligosaccharides into the sarcoplasm after invasion by *Trichinella*.

Days post invasion	Our findings	Predicted carbohydrate structure
	↑SNA, unchanged level of ST6Gal1	SiA-α-2,6-Gal-β-1,4-GlcNAc 6`-sialyl lactosamine
10 d.p.i.	↑SNA, ↑PNA-Neu, unchanged level of ST6GalNAc2	SiA-α-2,6-Gal-β-1,3-GalNAc-α-1-Ser/Thr Gal-β-1,3-GalNAc(SiA-α-2,6-)-α-1-Ser/Thr
	↑SNA, unchanged level of ST6Gal1	SiA-α-2,6-Gal-β-1,4-GlcNAc 6`-sialyl lactosamine
14-40 d.p.i.	↑SNA, ↑PNA-Neu, ↑ST6GalNAc2	SiA-α-2,6-Gal-β-1,3-GalNAc-α-1-Ser/Thr Gal-β-1,3-GalNAc(SiA-α-2,6-)-α-1-Ser/Thr
	↑SNA, ↑VVL-B4-Neu, ↑HPA-Neu, ↑ST6GalNAc1	SiA-α-2,6-GalNAc-α-1-Ser/Thr Sialyl-Tn-Ag

HPA – *Helix pomatia* agglutinin, PNA – *Arachis hypogea* agglutinin, SNA – *Sambucus nigra* agglutinin, VVL-B_4_ – *Vicia villosa* lectin B_4_, Neu – neuraminidase, SiA – sialic acid, Gal – galactose, GalNAc – N-acetyl-D-galactosamine, GlcNAc – N-acetyl-G-glucosamine, Ser – serine, Thr – threonine, ST6Gal1, ST6GalNAc1, and -2 – sialyltransferases.

The staining with SNA, and PNA after neuraminidase treatment at day 10 p.i. indicate the presence of the structures SiA-α-2,6-Gal-β-1,3-GalNAc-α-1-Ser/Thr and Gal-β-1,3-GalNAc(SiA-α-2,6-)-α-1-Ser/Th. The staining with SNA could be due to accumulation of 6`-sialyllactosamine (SiA-α-2,6-Gal-β-1,4-GlcNAc), too. Between days 14 and 40,p.i. synthesis of sialyl-Tn-Ag (SiA-α-2,6-GalNAc-α-1-Ser/Thr)is also taken into consideration because of the intense staining with SNA, and VVL-B4 and HPA after neuraminidase treatment, and the newly appeared increasing expression of ST6GalNAc1 enzyme.

## 4 Discussion

Apart from the broad knowledge about the extracellular proteoglycan components [30], most of the information concerning glycosylation of skeletal muscle fibre is related to inherited or congenital diseases [31, 32] with little known about the normal oligosaccharide composition and glycoproteome. Cell surface glycosylation during muscle cell differentiation [33] and a description of the human muscle glycophenotype [34] were recently reported. The authors showed that distinct sets of muscle glycoproteins are selectively reactive towards different plant lectins, known to share same glycan specificity, and thus illustrated the great and still obscure diversity of skeletal muscle glycans – a diversity that can be even greater in disease conditions.

The extensive biological role of the sialic acids in all types of cellular communication processes had been thoroughly discussed [4, 5]. The sialylation of the glycoproteins in skeletal muscle tissue is not well investigated, even though the essential role of the sialic acids for the proper muscular function has been proven by many researchers [35, 36, 37, 38, 39, 40].Our work aimed to describe in detail the phenomenon of increased intracellular accumulation of α-2,6-sialylated glycoproteins in skeletal muscles, invaded by *Trichinella spiralis* – a parasitic nematode, expressing an unique virus-like behavior [41].

Two studies revealed a contradictory significance of the α-2,6-linked sialic acid for the process of apoptosis in cells of different histological origin [42, 43]. The mechanisms of apoptosis that take place in the portion of occupied sarcoplasm at the beginning of infestation by *Trichinella* have been already studied in detail [29, 44, 45]. In the time course of muscle infestation, the satellite cells are activated; they proliferate and differentiate into cytoplasm of the newly formed Nurse cell that persists for years [2]. The results from our study assumed accumulation of sialyl-Tn-Ag, 6`-sialyl lactosamine, SiA-α-2,6-Gal-β-1,3-GalNAc-α-Ser/Thr and Gal-β-1,3-GalNAc(SiA-α-2,6-)-α-1-Ser/Thr oligosaccharide structures into the occupied sarcoplasm. These structures, however, do not seem to be associated with the process of apoptosis that is triggered at the beginning of infestation because they persist just as intensely within the Nurse cell. On the other hand, *Trichinella spiralis* does not synthesize sialic acids [18, 46, 47, 48]; therefore the parasite origin of these glycans seems to be unlikely.

The concluded identification of sialyl-Tn antigen structure (Sia-α-2,6-GalNAc-α-1-Ser/Thr) is particularly interesting because of the appearance of ST6GalNAc1 mRNA after day 10 p.i., accompanied by detected staining with VVL-B_4_ after neuraminidase treatment at day 14 p.i. and thereafter. Transient protein expression of ST6GalNAc1 was also demonstrated in mouse skeletal muscle tissue sections only at day 14 p.i. with *T. spiralis* [49]. *In vitro* studies have shown that the human analogues of ST6GalNAc1 and 2 have similar activities when Tn antigen (GalNAc-α-1-Ser/Thr) alone is available. Whenever both Tn and T antigens (Gal-β-1,3-GalNAc-α-1-Ser/Thr) are present, ST6GalNAc1 acts preferentially on Tn antigen, whereas the ST6GalNAc2 acts preferentially on T antigen [26]. The binding specificities of PNA towards T antigen and of VVL-B_4_ towards Tn antigen are well investigated [19, 23]. The expression levels of ST6GalNAc2 were relatively high in control muscle sample and increased after invasion with *Trichinella*, besides the staining of the invaded sarcoplasm with PNA after neuraminidase pretreatment was well distinguishable even at day 10 p.i. Therefore, we concluded that the expression of ST6GalNAc1 indicates synthesis of sialyl-Tn antigen structure. Expression of this sialyltransferase is absent in healthy mouse skeletal muscles [50], however our findings strongly suggest a new activation of ST6GalNAc1 gene in the occupied portions of skeletal muscle fibers, possibly triggered by excretory/secretory products of *Trichinella*, localized in the nuclei of the invaded muscles [51].

A recent study by Marini et al. [52] described histological expressions of sialic acids in human adult skeletal muscles. However, the only muscle glycoprotein known to be sialylated so far is the α-dystroglycan, bearing α-2,3-sialylated oligosaccharide [53].

In summary, our findings provide initial and basic description of a topic, which had not been investigated so far – the structure of the α-2,6-sialylated glycans and their role in the biology of the skeletal muscle tissue. In addition, for first time we describe the phenomenon of intracellular accumulation of these glycans into the developing Nurse cell after invasion of the muscles by the parasitic nematode *Tichinella*. Investigations in this domain will develop the understanding about the amazing adaptive capabilities of the skeletal muscle tissue.
